# Factors contributing to exercise tolerance in patients with coronary artery disease undergoing percutaneous coronary intervention

**DOI:** 10.1186/s13102-023-00640-4

**Published:** 2023-03-20

**Authors:** Husheng Li, Minqian Wei, Lili Zhang, Lan Huang, Yiyan Wang, Jiaqi Wang, Shaowei Zhuang, Xubo Wu, Jing Wu

**Affiliations:** 1 School of Nursing, Shanghai University of Traditional Chinese Medicine, No. 1200, Cailun Road, Pudong New District, Shanghai, 201203 China; 2 Department of Nursing, Shanghai Chest Hospital, Shanghai Jiao Tong University School of Medicine, Shanghai, 200030 China; 3 Department of Rehabilitation Therapy, Shanghai Seventh People’s Hospital, No. 358, Datong Road, Pudong New District, Shanghai, 200137 China; 4 Department of Cardiology, Shanghai Seventh People’s Hospital, Shanghai, 200137 China

**Keywords:** Exercise tolerance, Cardiopulmonary exercise testing, Percutaneous coronary intervention, Coronary artery disease, Cardiac rehabilitation

## Abstract

**Background:**

Exercise tolerance plays a vital role in the process of cardiac rehabilitation in patients undergoing percutaneous coronary intervention (PCI). The study sought to determine the characteristics, risks and correlates of post-PCI exercise tolerance in patients with coronary artery disease (CAD).

**Methods:**

We analyzed clinical data of 299 CAD patients undergoing elective PCI and completing cardiopulmonary exercise testing (CPET). According to the Weber classification, post-PCI exercise tolerance was evaluated by peak oxygen uptake (VO_2_ peak). We assessed the impact of 34 predefined clinical features, cardiac functional parameters, and blood biochemistry data on exercise tolerance by univariate analysis and logistics regression analysis.

**Results:**

Of 299 patients, 74.92% were men and average age was 60.90 ± 10.68 years. VO_2_ peak in the entire population was 17.54 ± 3.38 ml/kg/min, and 24.41% (*n* = 73) were less than 16 ml/kg/min, who were considered to have exercise intolerance. Multivariate logistics regression results showed that sex, diabetes mellitus, number of stents, left atrial diameter (LAD), end-diastolic volume (EDV), and hemoglobin influenced the peak oxygen uptake of CAD patients undergoing elective PCI. (All *p* < 0.05).

**Conclusions:**

Nearly one quarter of CAD patients have exercise intolerance in the early post-PCI period. Female, diabetes mellitus, number of stents, LAD, EDV might negatively impacted post-PCI exercise tolerance, which need further warrant by large scale cohort study.

**Supplementary Information:**

The online version contains supplementary material available at 10.1186/s13102-023-00640-4.

## Introduction

Coronary artery disease (CAD) is the leading cause of mortality and loss of disability worldwide [1]. Patients with CAD suffers significant symptoms of ischemia and hypoxia, due to the insufficient coronary flow and pressure that rise to meet the demands of physical activity. Percutaneous coronary intervention (PCI) is the priority treatment for CAD patients because it can rapidly unblock the infarct-related artery, restore myocardial perfusion, and reduce the infarct size [2, 3]. Even though, CAD patients undergoing PCI might experience symptomatic complications overtime, such as dyspnea, palpitations, dizziness on exertion. Evidence from epidemiological survey demonstrated that over half of post-PCI patients with the confession of exercise intolerance or muscular fatigue often get overlooked in clinical setting [4, 5].

Exercise tolerance [6, 7] referred to the maximum aerobic exercise capacity that can be tolerated without morbid symptoms and/or medical signs, and represents the body’s ability to absorb oxygen. Peak oxygen uptake (VO_2_ peak), measured during the cardiopulmonary exercise test (CPET), is the body’s maximum capacity to deliver and utilize oxygen, and is also the gold standard for assessment of exercise tolerance [8]. It can predict reinfarction and all-cause death in CAD patients and was used for prognosis assessment [9–11]. Previous studies [12–14] have reported that factors contributing to exercise tolerance include age, sex, body mass index (BMI), fasting blood glucose, ejection fraction, as well as nephropathy and peripheral arterial disease, etc. However, few existing researches have focused on the exercise tolerance status among PCI patients.

We hypothesized that, in addition to demographic and disease factors, measures of cardiac function and blood biochemistry would predict exercise tolerance. The identification of predictors of exercise tolerance may help improve future study designs by revealing confounding variables, as well as providing a theoretical basis for future cardiac rehabilitation schemes for patients. Therefore, this study aimed to describe the current status of exercise tolerance as well as to identify its predictors in CAD patients undergoing PCI.

## Methods

### Participants

The study population consisted of CAD patients undergoing elective PCI for stable angina from January 2019 to December 2020. All subjects complete a maximal symptom limited, incremental CPET 1 month after PCI. Patients without contraindications were routinely treated with secondary prevention drugs such as dual antiplatelet agents, statins, angiotensin-converting enzyme inhibitors (ACEI)/angiotensin receptor blocker (ARB) and β-blockers postoperatively. Patients were excluded from this study if they met any of the following: (1) combination of other major systemic diseases, such as mid- to late-stage tumors, liver disease, renal disease and pulmonary impairment; (2) incomplete medical records; (3) presence of contraindications to CPET, including unstable angina, acute myocardial infarction 3–5 days, arrhythmias associated with unstable hemodynamic disturbances, active myocarditis or pericarditis, aortic stenosis, heart failure or pulmonary embolism, unstable lower extremity venous thrombosis, moderate to severe asthma, in the acute phase of infection, suffering from abnormal psychiatric symptoms or physical disability.

### Interventional procedure

All patients provided explicit written informed consent prior to undergoing cardiac catheterization. Antiplatelet therapy was given to all patients before PCI with a specific regimen of Aspirin 100 mg once daily and Clopidogrel 75 mg once daily (or Tegretol 90 mg twice daily). Glycoprotein IIb/IIIa inhibitors were administered during the procedure and immediately after PCI, at the surgeon's discretion. The choice of coronary stent type and other adjuvant therapy is at the discretion of the primary surgeon, with a complete shift to drug-eluting stents in recent years. All stents were implanted at moderate to high deployment pressures (12–16 atm). Routine anticoagulation with low molecular heparin was continued postoperatively.

### Measurements

Sociodemographic characteristics, medical and medication history, CPET parameters, echocardiographic parameters, and laboratory data were collected from participants’ medical records and interviews. The investigation conforms to the principles outlined in the Declaration of Helsinki [15]. The study was reviewed and approved by the Human Study Committee of Shanghai Seventh People’s Hospital (Registration No. 2021-7th-HIRB-012), and informed consent was formally obtained from each participant.

#### Cardiopulmonary exercise testing

Maximal symptom limited, incremental CPET was performed using cycle ergometers (Quark PFT Ergo, COSMED, Rome, Germany) with a ramp protocol [16, 17]. Before the test begins, the clinician will conduct a comprehensive evaluation and formulate an appropriate exercise increment plan for the patient. The exercise protocol started with a 3-min resting phase on the cycle ergometer, followed by a 3-min warm-up phase at 20-Watt initial workloads. Then, workload was set at 35 Watt followed by an increase of 10–30 Watt increments per min at pedaling speed > 60 rpm until the patient has exhaustion or restrictive symptoms or signs [18], e.g., reaching the submaximal heart rate; respiratory exchange rate (RER) ≥ 1.0; electrocardiogram ST segment changes, etc. The following recovery phase consisted of 2-min active recovery at 20 Watt at pedalling speed between 50 and 60 rpm, followed by 3-min passive recovery.

During the whole test process, clinicians pay attention to monitoring the patient’s real-time ECG, blood pressure, gas exchange parameters, etc. The test was terminated when the subject showed one of the following conditions: (1) chest pain, dyspnea, pallor, weakness, dizziness, lower extremity pain, or unsteadiness in standing and requested to terminate; (2) ECG suggestive of myocardial ischemia; (3) II- or III-degree atrioventricular block; (4) systolic blood pressure decreased > 20 mmHg; (5) hypertension: systolic blood pressure > 250 mmHg; diastolic blood pressure > 120 mmHg; (6) rating of perceived exertion (RPE) up to Borg 19–20.

CPET core indicators such as VO_2_ peak, oxygen uptake efficiency slope (OUES), ventilatory efficiency (VE/VCO_2_) slope, etc. were measured. According to the Weber classification [19], VO_2_ peak < 16 ml/kg/min was considered to have objective exercise intolerance.

#### Echocardiographic examination

A Vivid E9 Color Doppler Ultrasound System with a 3.4 MHz transducer (GE Ultrasound, Horten, Norway) was used to conduct standard transthoracic 2D echocardiography. Exploring the parasternal long-axis view of the left ventricle can measure the thickness of the interventricular septum (IVST), the end-diastolic diameter of the left ventricle (LVDd), the end-systolic diameter of the left ventricle (LVDs), and the left atrial diameter (LAD). In the apical four-chamber view and the two-chamber view, the left ventricular ejection fraction (LVEF) was calculated using the Simpson formula. Pulse-Doppler can detect the blood flow spectrum of the mitral valve in the apical four chambers, and measure the double peaks of the mitral valve during diastole. The images generated by the echocardiography are all gathered and kept in the instrument's hard disk by two qualified cardiology fellows.

#### Laboratory testing

All measurements were performed in a central laboratory. Blood hemoglobin and platelet were automatically assessed using high-volume hematology analyzer Siemens Advia 2120 (Siemens Healthcare Diagnostics, Deerfield, IL, USA). Homocysteine, serum creatinine (Scr), uric acid (UA), and lipid profile [blood total cholesterol (TC), triglyceride concentrations (TG), high-density lipoprotein cholesterol (HDL-C), and low-density lipoprotein cholesterol (LDL-C)] were automatically assessed on Roche Cobas 8000 (Roche Diagnostics International Ltd, Rotkreuz, Switzerland).

### Statistical analysis

Data were analyzed using the Statistical Package for the Social Sciences ver. 24.0 (SPSS Inc., Chicago, IL, USA). One-sample K–S normality tests were performed on the measurement data. Normally distributed data were expressed as Mean ± SD and compared using the independent samples *t*-test; skewed data were expressed as median (interquartile range) and compared using the Mann–Whitney *U* test. The categorical variables were described as number of cases, and comparisons were performed by Chi-squared or Fisher analysis. The level of statistical significance was set at a *p*-value less than 0.05.

One-dimensional linear regression was used to assess the correlation between VO_2_ peak/Kg and selected variables. Logistic regression was performed to investigate significant predictors to identify exercise intolerance. The independent variables relevant to the logistic regression model were selected from the univariate analysis of demographic and procedural characteristics and clinical indicators, based on a threshold *p*-value of 0.05.

## Results

**Fig. 1:**
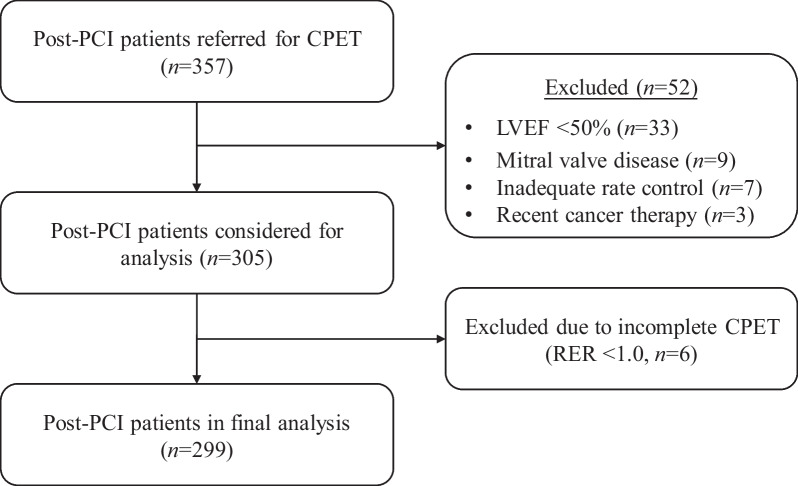
CONSORT diagram of study recruitment. *PCI* percutaneous coronary intervention, *LVEF* Left ventricular ejection fraction, *CPET* Cardiopulmonary exercise testing

We finally recruited 299 consecutive post-PCI patients. Figure 1 presents the flow diagram for the recruitment and analysis. Demographic and procedural characteristics were summarized in Table 1. Of the 299 patients, 74.92% were male and the average age was 60.90 ± 10.69 years. The most frequent comorbidity was hypertension (88.63%), followed by dyslipidemia (73.24%) and diabetes mellitus (24.75%). The comparison between the two groups indicated that sex, BMI, number of stents, prevalence of dyslipidemia, and diabetes mellitus were statistically significant. No significant difference in drug categories and lesion vessel were observed between groups.

**Table 1:** Comparison of demographic and procedural characteristics

Variables	All patients (*n* = 299)	Exercise tolerance (*n* = 226)	Exercise intolerance (*n* = 73)	*p*-value
Mean age (year)	60.90 ± 10.68	60.47 ± 10.49	62.45 ± 11.24	0.217
Male, *n* (%)	224 (74.92)	180 (79.65)	44 (60.27)	0.001
BMI (kg/m^2^)	25.68 ± 3.39	25.33 ± 3.12	26.73 ± 3.94	0.002
Comorbidity, *n* (%)				
Hypertension	265 (88.63)	198 (87.61)	67 (91.78)	0.329
Dyslipidemia	219 (73.24)	159 (70.35)	60 (82.19)	0.047
Diabetes mellitus	74 (24.75)	46 (20.35)	28 (38.36)	0.002
No. of stents, *n* (%)				< 0.001
1	79 (26.42)	65 (28.76)	14 (19.18)	
2	160 (53.51)	131 (57.96)	29 (39.73)	
3	42 (14.05)	25 (11.06)	17 (23.29)	
≥ 4	18 (6.02)	5 (2.21)	13 (17.81)	
Lesion vessel, *n* (%)				
LAD	191 (63.88)	139 (61.50)	52 (71.23)	0.132
LCX	107 (35.79)	77 (34.07)	30 (41.10)	0.276
LM	37 (12.37)	27 (11.95)	10 (13.70)	0.693
RCA	146 (48.83)	110 (48.67)	36 (49.32)	0.924
Medication, *n* (%)				
Aspirin	289 (96.66)	218 (96.46)	71 (97.26)	0.741
Clopidogrel	251 (83.95)	190 (84.07)	61 (83.56)	0.918
Statins	277 (92.64)	209 (92.48)	68 (93.15)	0.848
ACEI/ARB	195 (65.22)	146 (64.60)	49 (67.12)	0.694
Calcium antagonist	99 (33.11)	72 (31.86)	27 (36.99)	0.418
β-blockers	237 (79.26)	174 (76.99)	63 (86.30)	0.088
Nitrates	238 (79.60)	178 (78.76)	60 (82.19)	0.527

*LAD* Left anterior descending artery, *LCX* Left circumflex branch artery, *LM* Left coronary artery main stem, *RCA* Right coronary artery, *BMI* Body mass index, *ACEI* Angiotensin-converting enzyme inhibitors, *ARB* Angiotensin receptor blocker

**Fig. 2:**
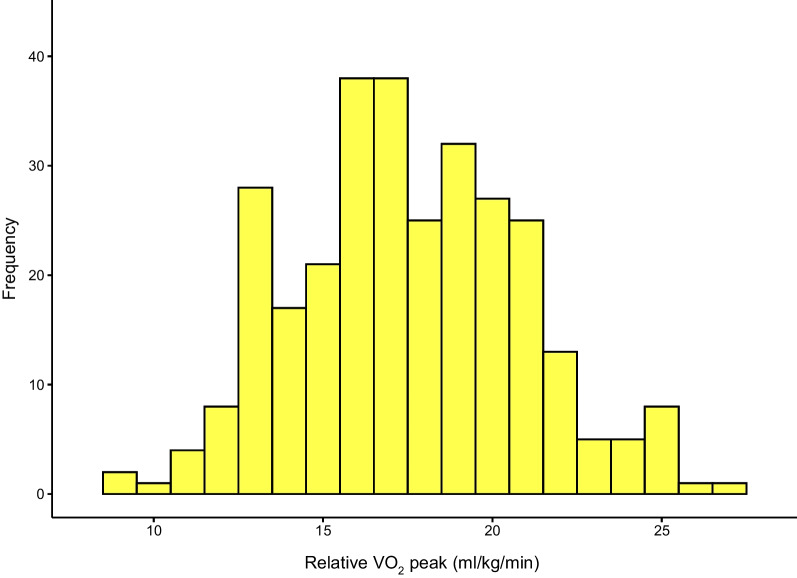
Distribution of peak oxygen consumption (VO_2_ peak/Kg) among entire cohort

Six patients were limited by musculoskeletal pain and failed to complete the CPET protocol and achieve a maximal effort. All remaining 299 subjects who completed CPET had no major cardiac events, ischemic ECG changes or sustained ventricular arrhythmias during the testing period. VO_2_ peak in the entire population was 17.54 ± 3.38 ml/kg/min, and 24.41% (*n* = 73) were less than 16 ml/kg/min, considered to have objective exercise intolerance. The distribution of VO_2_ peak is shown in Fig. 2, and the remaining core indicators of CPET were listed in Additional file 1: Table S1. In contrast, patients in exercise intolerance group were more likely to have decreased peak heart rate, VO_2_ peak, METs, and OUES, whereas VE/VCO_2_ slope and HRR Max showed increasing trend.

Table 2 shows the differences in clinical features. In terms of transthoracic echocardiographic variables, LAD and EDV were higher in exercise intolerance group. As for blood biochemistry data, hemoglobin was 138.10 ± 14.13 g/l in the overall population and higher in normal exercise tolerance group. (All *p* < 0.001).

**Table 2:** Comparison of clinical indicators

Variables	All patients (*n* = 299)	Exercise tolerance (*n* = 226)	Exercise intolerance (*n* = 73)	*p*-value
*Transthoracic echocardiography*				
LAD (mm)	34.46 ± 4.31	33.79 ± 4.05	36.55 ± 4.46	< 0.001
LVDd (mm)	45.12 ± 6.11	44.96 ± 5.71	45.60 ± 7.24	0.436
LVDs (mm)	29.73 ± 4.68	29.63 ± 4.07	30.04 ± 6.21	0.518
IVST (mm)	10.75 ± 3.38	10.77 ± 3.81	10.67 ± 1.34	0.821
LVEF (%)	63.77 ± 6.52	63.70 ± 6.27	63.98 ± 7.26	0.754
EDV (ml)	98.16 ± 26.53	95.04 ± 23.14	107.80 ± 33.35	< 0.001
FS (%)	34.94 ± 4.84	35.07 ± 4.63	34.54 ± 5.44	0.414
*Blood biochemistry*				
Hemoglobin (g/l)	138.10 ± 14.13	139.87 ± 13.77	132.64 ± 13.94	< 0.001
Platelet (× 10^9^/l)	209.07 ± 60.23	206.27 ± 53.12	217.81 ± 77.87	0.155
Homocysteine (μmol/l)	13.82 ± 4.48	13.88 ± 4.15	13.62 ± 5.41	0.666
Scr (umol/l)	65.11 ± 16.22	65.34 ± 14.69	64.41 ± 20.35	0.673
UA (umol/l)	351.42 ± 93.44	356.51 ± 91.53	335.67 ± 98.11	0.098
TC (mmol/l)	3.91 ± 1.06	3.95 ± 1.04	3.77 ± 1.13	0.192
TG (mmol/l)	1.92 ± 1.64	1.95 ± 1.76	1.84 ± 1.19	0.615
HDL-C (mmol/l)	1.07 ± 0.26	1.08 ± 0.26	1.03 ± 0.26	0.160
LDL-C (mmol/l)	2.31 ± 0.93	2.33 ± 0.92	2.25 ± 0.96	0.545

*LAD* Left atrial diameter, *LVDd* Left ventricular diastolic diameter, *LVDs* Left ventricular systolic diameter, *IVST* Thickness of the interventricular septum, *LVEF* Left ventricular ejection fraction, *EDV* End-diastolic volume, *FS* Fractional shortening, *Scr* Serum creatinine, *UA* Uric acid, *TC* Total cholesterol, *TG* Triglyceride concentrations, *HDL-C* High-density lipoprotein cholesterol, *LDL-C* Low-density lipoprotein cholesterol

**Fig. 3:**
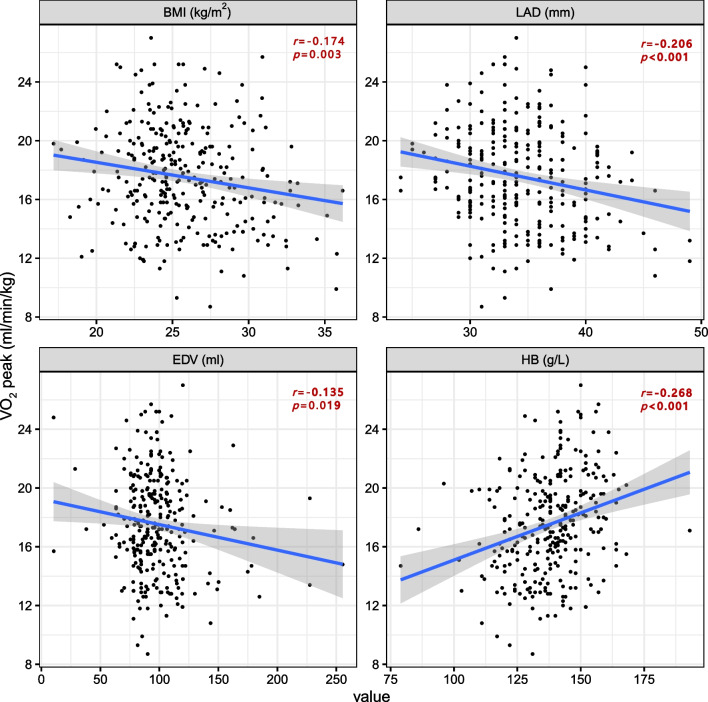
Regression plots between VO_2_ peak/Kg, BMI, LAD, EDV and hemoglobin. *BMI* Body mass index, *LAD* Left atrial diameter, *EDV* End-diastolic volume

The linear regression plots between BMI, LAD, EDV, hemoglobin and VO_2_ peak/Kg were shown in Fig. 3. VO_2_ peak/Kg was inversely related to BMI (*r* = − 0.174,* p* = 0.003), LAD (*r* = − 0.206,* p* < 0.001) and EDV (*r* = − 0.135,* p* = 0.019), whereas it was positively correlated with hemoglobin (*r* = 0.268,* p* < 0.001). Furthermore, logistic regression models were performed to identify exercise intolerance, as presented in Table 3. Although dyslipidemia showed a trend towards an adverse effect on exercise capacity, it did not reach statistical significance (*p* = 0.054). Increased BMI (*OR* = 1.128, *p* = 0.003) was significantly associated with exercise intolerance in the univariate regression analysis; however, this association did not persist in the multivariate regression. Stepwise multivariate logistic analyses revealed that the number of stents (*OR* = 4.078, *p* < 0.001), diabetes mellitus (*OR* = 2.138, *p* = 0.027), LAD (*OR* = 1.173, *p* < 0.001), and EDV (*OR* = 1.199, *p* = 0.003) were linked to a higher risk of exercise intolerance, while being male (*OR* = 0.328, *p* = 0.003) and having a higher hemoglobin content (*OR* = 0.705, *p* = 0.006) were protective factors against exercise intolerance.

**Table 3:** Binary logistic regression analysis to identify exercise intolerance

Variables	Univariate regression analysis			Multivariate regression analysis		
	OR	95% CI	*p*-value	OR	95% CI	*p*-value
Sex			< 0.001			0.003
Male	0.388	0.219–0.685		0.328	0.156–0.692	
Female	Reference			Reference		
No. of stents			< 0.001			< 0.001
> 2	4.558	2.491–8.340		4.078	2.060–8.071	
≤ 2	Reference			Reference		
Diabetes mellitus			0.002			0.027
Yes	2.435	1.374–4.315		2.138	1.091–4.189	
No	Reference			Reference		
Dyslipidemia			0.054			
Yes	1.942	0.097–3.778				
No	Reference					
BMI (kg/m^2^)	1.128	1.043–1.220	0.003	Not entered		–
LAD (mm)	1.165	1.091–1.245	< 0.001	1.173	1.081–1.272	< 0.001
EDV (per 10 ml)	1.188	1.072–1.316	0.001	1.199	1.066–1.349	0.003
Hemoglobin (per 10 g/l)	0.691	0.567–0.842	< 0.001	0.705	0.549–0.905	0.006

Factors found significant in univariate regression analysis were included in a forward stepwise multivariate logistics regression model with included criteria of *p* < 0.05 and removal criteria of *p* > 0.1. And Nagelkerke R^2^ for the multivariate model = 0.373

*OR* Odds ratio, *CI* Confidence interval, *Ref* Reference, *BMI* Body mass index, *LAD* Left atrial diameter, *EDV* End-diastolic volume

## Discussion

The VO_2_ peak is the most objective and reliable indicator to measure exercise tolerance in CAD patients [20, 21]. In our study, the average VO_2_ peak was found to be 17.54 ± 3.38 ml/kg/min, with nearly a quarter of the patients (73 cases, 24.41%) experiencing post-PCI cardiopulmonary dysfunction. These results suggest that even if coronary revascularization is successfully completed, exercise tolerance in the early postoperative period does not return swiftly to the normal level, which is in line with the findings of Li et al. [5]. Although PCI can improve symptoms of myocardial ischemia, myocardial contractile function and cardiac compliance remain abnormal in the early stages, resulting in reduced cardiac output and delayed recovery of exercise tolerance. Additionally, patients may opt not to exercise or exercise less due to concerns about wounds or myocardial infarction, or because they lack professional and scientific exercise planning guidance, all of which contributes to a lack of improvement in exercise tolerance in the early stages.

Our study found that men and patients with higher hemoglobin levels had better post-PCI exercise tolerance. As noted by Kodama et al. [22], men have higher cardiorespiratory fitness values, about 2 METs higher, compared to women of the same age. This disparity can be attributed to differences in anatomy and physiology, such as: (1) Women having smaller left ventricles and lower ejection volumes[23]; (2) Lower left ventricular diastolic compliance in women [24]; (3) A higher proportion of obesity in women [23]; (4) Women being more prone to iron deficiency and having lower hemoglobin levels compared to men [25, 26]. Hemoglobin is a main marker of anemia and a primary performer of red blood cell function, responsible for transporting and carrying oxygen and carbon dioxide within the red blood cells. Decreased hemoglobin levels can result in a further deterioration of the hemodynamic state. In addition, aging in elderly patients leads to an increase in underlying diseases, weakens the body's immune function, and makes them more prone to recurrent infections, all of which exacerbates the decline in post-PCI exercise tolerance.

Diabetes mellitus is a separate risk factor. According to Gürdal et al. [27], the VO_2_ peak and anaerobic threshold of diabetic patients were significantly lower than those in healthy adults. Plausibility of this mechanism is strengthened by several pathological pathways, such as microvascular disease, energy metabolism disorders, and autonomic dysfunction, which are independent of hypertension and coronary artery disease. These pathological changes result in ventricular diastolic dysfunction and impaired heart rate recovery, thereby impacting exercise tolerance [28–31].

With more stents implanted, CAD patients are at a higher risk of postoperative exercise intolerance, which is consistent with the findings of the SYNTAX trial [32]. The number of stents and the total length of the stents implanted are important indicators of the complexity of the coronary lesions and play a crucial role in predicting the clinical outcomes of patients undergoing PCI [33]. Studies have shown that an excessive number of stents can increase the damage to the endothelium during the procedure, exacerbating the local inflammatory response of the endothelium undergoing PCI [34, 35], which can trigger symptoms of exercise limitation.

LAD and EDV have been proposed as a morphophysiological marker of ventricular dysfunction. This study found that increased LAD and EDV are also risk factors for postoperative exercise intolerance in CAD patients. Previous research has shown that when ventricular dysfunction occurs, diastolic filling pressure increases, ventricular compliance decreases, left atrial pressure increases, pulmonary vein and capillary wedge pressure increases, and pulmonary ventilation perfusion is impaired. These changes result in elevated VE/VCO_2_, insufficient left ventricular filling, reduced cardiac output, shortness of breath during exertion, and decreased exercise tolerance [4, 36–38].

Interestingly, LVEF, a commonly used indicator, does not seem to predict exercise tolerance. One reason is that post-PCI patients with LVEF less than 50% are usually considered temporarily unfit for CPET and therefore do not have data for analysis in this study. On the other hand, the relationship between exercise capacity and LVEF may be impacted by diverse comorbidities, and various compensatory mechanisms can help preserve exercise ability [39]. Smart et al. [40] found that rest LVEF was weakly correlated with peak oxygen uptake, which was more closely related to a composite model filling pressure, systolic and diastolic function.

Patients with CAD tend to be sedentary undergoing PCI [41], which may lead to cardiorespiratory deconditioning as well as muscle atrophy and weakness that in turn leads to deterioration in metabolic, cardiorespiratory, and functional health. However, increasing physical activity through comprehensive cardiac rehabilitation can improve exercise tolerance and quality of life in these patients [42–44]. Meta-analyses reported that endurance and resistance training together increased peak oxygen uptake and 6-min walk test distance by 2.2 ml/kg/min and 33 m, respectively [45, 46]. It has been noted that an increase in cardiorespiratory fitness is associated with a reduction in the risk of all-cause mortality and cardiovascular mortality [22]. Thus, it is imperative that medical staff provide early exercise guidance and health education to CAD patients undergoing PCI, and offer personalized exercise rehabilitation programs to enhance their post-PCI exercise tolerance.

## Limitations

As an observational study, it has a limited number of included cases and may have problems such as selection bias. It is necessary to further expand the sample size for prospective study design. Enrollment was limited by the prescription of CPET, patients who could not tolerate CPET were not included in the study, and patients with more severe disease were excluded. None of the included patients discontinued β-blockers during CPET, which had a certain impact on the study results. Compared with the treadmill exercise program, the peak oxygen uptake for cycle ergometer exercise program was reduced by about 10–20%, so the result of the CPET index was low.

## Conclusions

Our main finding revealed that nearly a quarter (73 cases, 24.41%) of CAD patients have exercise intolerance in the early post-PCI period. Sex, the number of stents, diabetes mellitus, LAD, EDV and hemoglobin were identified as independent factors contributing to exercise intolerance. It is recommended to further explore a comprehensive cardiac rehabilitation model including exercise rehabilitation, symptom management and weight management, in order to improve the post-PCI exercise tolerance and relieve postoperative discomfort of CAD patients.

## Supplementary Information

**Additional file 1**.** Table S1**: Comparison of CPET core indicators.

## Data Availability

The dataset used and analyzed during the current study is available from the corresponding author on reasonable request.

## Abbreviations

ACEI: Angiotensin-converting enzyme inhibitors
ARB: Angiotensin receptor blocker
BMI: Body mass index
CAD: Coronary artery disease
CI: Confidence interval
CPET: Cardiopulmonary exercise test
HDL-C: High-density lipoprotein cholesterol
IVST: Interventricular septum
LAD: Left atrial diameter
LDL-C: Low-density lipoprotein cholesterol
LVDd: End-diastolic diameter of the left ventricle
LVDs: End-systolic diameter of the left ventricle
LVEF: Left ventricular ejection fraction
OR: Odds ratio
OUES: Oxygen uptake efficiency slope
PCI: Percutaneous coronary intervention
RER: Respiratory exchange rate
RPE: Rating of perceived exertion
Scr: Serum creatinine
TC: Total cholesterol
TG: Triglyceride concentrations
UA: Uric acid
VE/VCO_2_ slope: Ventilatory efficiency slope
VO_2_ peak: Peak oxygen uptake
