# Supplementary material for: Factors contributing to exercise tolerance in patients with coronary artery disease undergoing percutaneous coronary intervention
Source: BMC Sports Sci Med Rehabil. 2023 Mar 20;15:35. doi: 10.1186/s13102-023-00640-4 (PMC10026462; doi:10.1186/s13102-023-00640-4)
Supplement: Supplementary file 1 — Additional file 1. Table S1: Comparison of CPET core indicators. [file 13102_2023_640_MOESM1_ESM.docx]

**Table S1 Comparison of** **CPET** **core indicators**

| Variables | All patients  (*n*=299) | Exercise tolerance (*n*=226) | Exercise intolerance (*n*=73) | *p*-value |
| --- | --- | --- | --- | --- |
| VO_2_ peak (ml/min/kg) | 17.54±3.38 | 18.94±2.57 | 13.22±1.32 | ＜0.001 |
| VO_2_ peak (L/min) | 1.26±0.31 | 1.35±0.29 | 0.98±0.20 | ＜0.001 |
| Peak heart rate (bpm) | 121.01±14.77 | 123.59±14.15 | 113.01±13.87 | ＜0.001 |
| Peak VO_2_/HR (ml/beat) | 10.50±2.43 | 11.03±2.34 | 8.83±1.93 | ＜0.001 |
| Peak METs | 5.02±1.00 | 5.39±0.73 | 3.86±0.83 | ＜0.001 |
| VE/VCO_2_ slope | 27.37±4.26 | 26.94±3.74 | 28.71±5.38 | 0.002 |
| OUES (ml/logL) | 1788.58±424.53 | 1886.42±404.22 | 1485.67±335.03 | ＜0.001 |
| HRR 1 minute (beat) | 18.30±7.56 | 19.13±7.46 | 15.67±7.31 | 0.001 |
| HRR Max (beat) | 40.07±17.47 | 37.31±14.05 | 48.60±23.43 | ＜0.001 |

Abbreviations: VO_2_ peak: peak oxygen uptake; VO_2_/HR: oxygen pulse; METs: metabolic equivalents/ exercise capacity; VE/VCO_2_ slope: ventilatory efficiency slope; OUES: oxygen uptake efficiency slope; AT: anaerobic threshold; HRR 1 minute: heart rate recovery at 1 minute after exercise; HRR Max: maximal heart rate reserve.
